# Ultra-Low-Cross-Linked Microgels Reveal Unexpected Dynamics in Overcrowded Conditions

**DOI:** 10.1021/acsmacrolett.5c00787

**Published:** 2026-02-18

**Authors:** Nikolaos A. Burger, Alexander V. Petrunin, Ann E. Terry, Andrea Scotti

**Affiliations:** † Division of Physical Chemistry, 5193 Lund University, SE-22100 Lund, Sweden; ‡ Institute of Physical Chemistry, RWTH Aachen University, 52056 Aachen, Germany; § MAX IV Laboratory, Lund University, P.O. Box 118, 22100 Lund, Sweden

## Abstract

Ultralow-cross-linked microgels serve as powerful model systems for investigating structure–rheology relationships in soft colloidal suspensions. Using precipitation polymerization, we obtain both self-cross-linked microgels with a weakly cross-linked core, surrounded by an ultrasoft corona (ULC), and regular cross-linked (RC) microgels. ULC microgel suspensions exhibit distinctive rheological responses in crowded conditions. Their linear viscoelastic behavior shares features with critical-like gels, characterized by *G*′ ∼ *G*″ ∼ ω^*n*^. Large-amplitude-oscillatory-shear measurements reveal a solid–liquid transition reminiscent of polymeric networks lacking a *G*″ overshoot during yielding. Stress-shear strain rate measurements further reveal shear-thinning with a power-law behavior at low shear strain rates, σ ∼ γ̇^∼0.25^. We attribute this behavior to a fine-tuned balance between polymeric and colloidal contributions. This rheological response to crowding establishes ULC microgels as emergent soft nanocolloids with potential biological relevance, particularly as analogues for the heterogeneity in mechanical softness (compressibility) observed in cell membranes.

## Graphical abstract

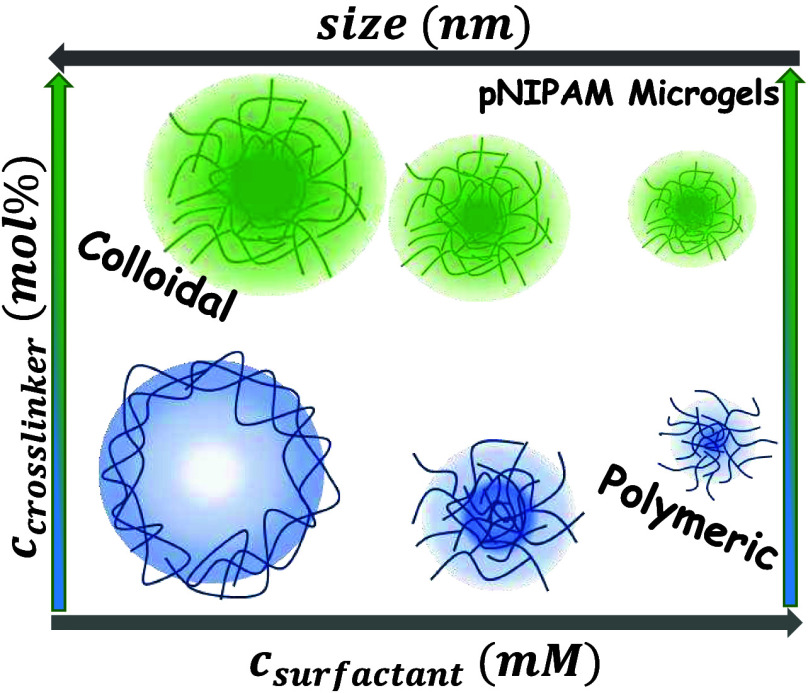

Concentrated suspensions of soft colloids are of broad interest in soft matter physics and materials science. Modifications to their internal architecture strongly affect the particle’s compressibility (softness),^1,2^ enabling fine-tuning of the balance between colloid and polymer contributions, with important consequences for both flow and phase behavior.^2–4^ Both the flow and phase behavior of colloidal suspensions containing particles characterized by a soft repulsive interaction potential differ significantly from those of incompressible hard-sphere suspensions.^5–7^ The effect of softness on the flow properties of soft colloidal suspensions has been extensively studied using star polymers^8^ but it remains less explored for other particle architectures, e.g., microgels.^9,10^ In this context, it is established that softness plays a crucial role in the functional processes of biological systems such as cell membranes and connective tissues.^11,12^ The stiffening of a cell is linked with a phase transition and mediated by colloids suspended within the cell, which is reminiscent of the glass transition observed for soft colloids interacting via a repulsive interaction potential.^13^ Colloidal aggregation within the hydrogel is reminiscent to the behavior of the cell and the aggregation process of RNA and proteins in the cytoplasm, which defines how the cytoplasm itself deforms and flows under stress.^14^ Even small changes within the cell can lead to significant alterations in the linear and nonlinear viscoelastic behavior of soft connective tissues, potentially impairing their functionality.^15,16^ Moreover, the mechanical properties and phase behavior of the cell membrane are influenced by the incorporation and transformations of soft, flexible proteins (e.g., protein folding or unfolding, and crystallization).^17^ While the softness of individual colloids within a cell is one factor that determines its overall mechanical behavior, given the biological complexity of the problems, it has not yet been possible to disentangle the roles of individual characteristics (e.g., interfacial charges, compressibility, shape, size) on these properties. Although repulsive systems present a simplified approach, they remain promising candidates for material engineering due to their tunable properties.^2,18^ Self-cross-linked microgels, i.e., microgels synthesized via precipitation polymerization in the absence of added cross-linker agent, herein referred to as ultra low-cross-linked (ULC) microgels, represent the softest class of synthetic colloids due to their very low bulk modulus, their extremely high swelling ratio, low molecular weight, and their response to crowding (decrease of nearest neighbor distance, NND).^2,19^

The concentration dependence of viscosity, η, for ULC microgel suspensions^19^ shows notable similarities to flexible polymer solutions and associative polymers, which form viscoelastic networks through topological constraints and entanglements without ever reaching dynamic arrest.^20–23^ Suspensions of hard colloids undergo a glass transition characterized by a dynamic arrest with increasing volume fraction ϕ.^5,24,25^ With respect to hard spheres, soft colloids can squeeze, so instead of ϕ, the use of a generalized packing fraction, ζ is appropriate (Section III in SI). Due to their ability to osmotically deswell, facet, and interpenetrate, soft colloids form glasses at much higher ζ ∼ 0.7 – 1.4.^2,3,26,27^ In this contribution, it is shown that ULC microgel suspensions in overcrowded environments (5.71 ≤ ζ ≤ 8.16), exhibit linear viscoelasticity compatible with critical-gels and charged colloids, where *G*′ ∼ *G*″ ∼ ω^*n*^.^28,29^ Large-amplitude-oscillatory-shear (LAOS) measurements reveal a solid–liquid transition reminiscent of polymeric networks with a lack of *G*″ overshoot and strain thinning behavior,^30^ whereas steady shear measurements (flow curves) reveal a universal power-law behavior of shear stress at low shear strain rate values, reminiscent of star polymer solutions.^31^ Moreover, for the ζ values studied here, the soft particles have already reached almost the maximum deswelling, as revealed by small-angle X-ray scattering (SAXS) measurements. Suspensions of regularly cross-linked (RC, synthesized by adding 3.7 mol % cross-linker agents) microgels instead undergo a glass transition at ζ < 1, as expected for soft colloids.^25,32^

We employed dynamic light scattering (DLS) and SAXS to characterize the dilute suspensions of microgels and establish their colloidal nature (across their volume phase transition temperature (VPTT; Figures S1 and S2). Details of the synthesis of microgels are described in SI, Section I.^19,33^ DLS reveals a hydrodynamic radius, *R*_H_, of 114 ± 2 and 107 ± 1 nm in the swollen state at *T* = 20 °C for ULC and RC microgels, respectively (Figure S1). ULC microgels have a higher swelling ratio, *S*_R_ = *R*_H_(20 °C)/*R*_H_(45 °C) ∼ 3.8, compared to RC microgels *S*_R_ ∼ 2, consistent with previous reports.^34,35^ *S*_R_ decreases with increasing cross-linker content *f*, following a power-law dependence.^35^ The amount of cross-links in the polymer network of the small ULC microgels studied here is estimated to be *f* ∼ 0.001. This value is approximately five times lower than the estimate value for larger ULC microgels (*f* ∼ 0.005) and about 20 times lower than that of RC microgels (*f* ∼ 0.02).^9,35^

The amount and possibly the type of surfactant define the final microgel size and internal architecture.^19,33,36^ Under cross-linker and surfactant-free conditions, the polymerization of NIPAM yields pNIPAM microgels with an unusual inverted cross-linking architecture, in which most of the polymer is cross-linked in the shell while the core remains relatively empty.^33,36,37^ The addition of surfactant (sodium dodecyl sulfate, SDS)^33,36^ leads to the formation of the expected core-fuzzy shell structure, characterized by a higher cross-link density in the core.^33^ Increasing SDS concentration, *c*_*SDS*_ during synthesis, induces changes in (i) size, with the microgel diameter decreasing, and (ii) softness, with the effective cross-link density decreasing, as evidenced by an increased swelling ratio.^19,35^ A gradual transition from a soft to an ultrasoft microgel is thus observed. This control is achieved by fine-tuning the ratio between the surfactants, the monomers, and the initiators as we describe in SI, Section I.^33,36,37^ Here, we focus on microgels with *c*_SDS_ = 2 mM. Figure 1 presents an out-of-scale schematic of ULC microgels synthesized at different SDS concentrations at *c*_SDS_ ∼ 0 mM (Figure 1(a)), *c*_SDS_ ∼ 0.4 mM (Figure 1(b)), and *c*_SDS_ ∼ 2 mM (Figure 1(c)).

**Figure 1:**
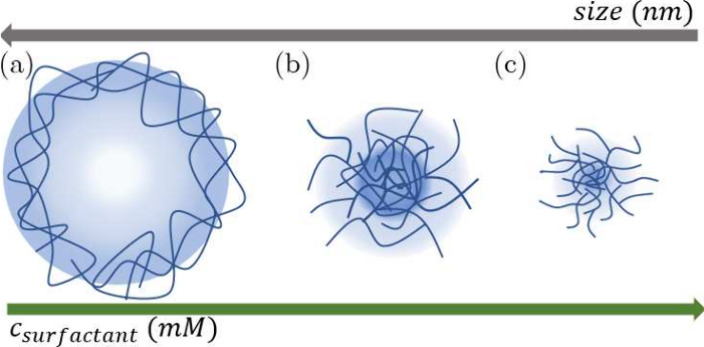
Out-of-scale structure of ULC microgels synthesized (a) without surfactant or cross-linker.^36^ (b) Low SDS concentration (*c*_SDS_ ∼ 0.4 mM).^9^ (c) High SDS concentration (*c*_SDS_ ∼ 2 mM).^19^

The SAXS curves further establish that the small ULC microgels, obtained from precipitation polymerization without the addition of any cross-linker agent, are microgels, not simple polymeric chains. Indeed, the oscillations of the scattering intensity, *I*, are clearly visible, and the fact that *I* drops with wavevector, *q*, with a power-law *I* ∼ *q* ^–4^ at intermediate *q* is a signature of a spherical object and not compatible with a polymeric coil (Figure S2). The radial distributions φ­(*R*) in Figure S2 obtained from the fits of the curves with a fuzzy-sphere model also show that the ULC microgels contain much less polymer within their volume compared to the RC. Figure 2(a) presents the frequency dependence of the loss tangent tan δ = *G*″/*G*′ (which describes the viscous relative to elastic contributions, where tan δ > 1 indicates liquid-like response, whereas tan δ < 1 solid-like), for both ULC and RC microgel suspensions. In the case of ULC microgels, the data exhibit a smooth transition from a liquid-like viscoelastic response at ζ = 3.06 ± 0.01 (up triangles) where tan δ = 1 at ω ∼ 10 rad/s and increases until reaches a plateau at low frequencies, typical behavior of maxwell fluids, to a solid-like viscoelastic response for ζ ≥ 5.71 ± 0.01, where tan δ < 1 and ω are independent.

**Figure 2:**
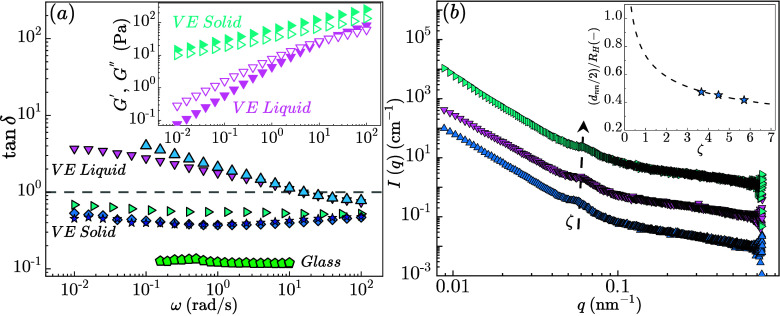
(a) Tan δ (normalized loss *G*″ to storage modulus *G*′) versus frequency, ω, for suspensions of ULC microgels at ζ = 3.67 ± 0.01 (up triangles), ζ = 4.48 ± 0.01 (down triangles), ζ = 5.71 ± 0.01 (right triangles), ζ = 7.03 ± 0.01 (diamonds), ζ = 8.16 ± 0.01 (stars) and RC ζ = 1.0 ± 0.04 (diamonds). Inset: *G*′ (filled) and *G*″ (open symbols) versus ω for ULC microgel suspensions at ζ = 4.48 ± 0.01 (down triangles) and ζ = 5.71 ± 0.01 (right triangles). (b) Small-angle X-ray scattering intensities *I*(*q*) versus scattering vector *q* of ULC microgels measured at 20 °C at 3.67 ± 0.01 (up triangles), 4.48 ± 0.01 (down triangles), 5.71 ± 0.01 (right triangles). The curves are shifted vertically by a factor of 10^1^ per ζ, from low to high ζ, for clarity. Inset: Normalized (with the hydrodynamic radius) nearest neighbor distance (*d*_nn_/2)/*R*_H_ versus ζ for ULC microgel suspensions.

For ζ ≥ 5.71 ± 0.01, the suspensions from viscoelastic liquids, turn into viscoelastic solids with *G*′ > *G*″ in the whole frequency domain. Both the storage and loss moduli exhibit power-law scaling with frequency, *G*′ ∼ *G*″ ∼ ω^*n*^ with *n* decreasing from *n* ∼ 0.3 ± 0.02 at ζ = 5.71 ± 0.01 to *n* ∼ 0.24 ± 0.02 at ζ = 8.16 ± 0.01 (inset of Figure 2 and Figure S3). These features in LVE of ULC microgel suspensions are compatible with the critical-gel dynamics observed in cross-linked polymers and charged soft colloids.^28,29^ The importance of the observed power-law behavior at high ζ is further highlighted if we consider that aging effects (evolution of *G*′, *G*″ with time) are not observed, as indicated by dynamic time sweep and repeatable dynamic frequency sweep measurements (Figure S4). Furthermore, the suspensions reach steady state immediately after shear cessation (inset of Figure S4), which implies that the reported scaling laws of *G*′, *G*″ with ω are time invariant. Given the scaling of *G*′, *G*″ with ω for different ζ values, we can consider ζ as the equivalent parameter to time, which is the key parameter in the case of cross-linked polymers.^28^

This scaling at elevated ζ is unusual for repulsive systems in crowded conditions, which makes the observed behavior very spectacular.^9,25,31^ Indeed, colloidal suspensions typically exhibit a pronounced divergence in viscosity and structural relaxation time as they approach the glass transition.^5^ In addition, the smooth transition observed from liquid-like to viscoelastic solid with power-law behavior (Figure S3) is also not characteristic of concentrated polymer solutions, as in that case the low-frequency regime is described by the Maxwell model.^22,23^

The position of the structural peak *q*_max_ from small-angle X-ray scattering (SAXS) reflects the average center-to-center distance or the nearest-neighbor distance, NND (*d*_nn_), between neighboring microgels: *d*_nn_ = 2π/*q*_max_. SAXS measurements exclude the possibility that the observed smooth transition of the viscoelastic response arises from significant further microgel deswelling, as the normalized (with the hydrodynamic radius) *d*_nn_ decreases slightly with ζ, as indicated in the inset in Figure 2(b).^27^ The solid line in the inset in Figure 2(b) follows the predictions of isotropic deswelling with *d*_nn_ = *c*ζ^–1/3^.^19^ The fact that the ratio, (*d*_nn_/2)/*R*_H_ is almost 0.4 indicates that the microgels have shrunk more than half their initial size, indicating crowding conditions.^26^

Our results can be explained considering that microgel–microgel interpenetration is most favorable compared to faceting, which is the expected case in RC microgels.^25^ In that case, the stress relaxation can be driven by arm retraction of the dangling ends and loose cross-linked regimes, resulting in the formation of a viscoelastic network instead of soft glass.

RC microgel suspensions exhibit a sequential transition from a liquid state to a viscoelastic solid and finally to a glassy state as ζ increases from ζ = 0.60 ± 0.03 to ζ = 0.80 ± 0.03, (Figure S3). The glass transition occurs at slightly higher ζ compared to hard spheres (ϕ = 0.58) due to the particle’s ability to deswell and facet, but it is in agreement with what is reported in the literature for cross-linked microgels with comparable softness.^2^ At ζ = 0.8 ± 0.03, the suspensions are characterized by a loss tangent tan δ = 0.1 that is frequency independent, with dominant storage modulus *G*′ ≫ *G*″ (Figure S3). The dimensionless analysis of frequency sweeps reveals that the viscoelastic response is influenced by the contributions of the soft polymeric shell of the individual microgels (Figure S5).

Large amplitude oscillatory shear (LAOS) experiments provide a robust framework for characterizing the linear and nonlinear viscoelastic response of these suspensions and offer critical insights into the underlying nonlinear phenomena.^30^ Figure 3 illustrates the dependence of *G*′, *G*″ on strain amplitude, γ (%), for ULC microgel suspensions at different ζ. *G*′ and *G*″ are normalized by the value in the linear viscoelastic regime, (*G*_γ=0.1%_^′^), (*G*_γ=0.1%_^″^), respectively. Figure S6 reports the raw data showing the solid–liquid transition upon increasing γ (%) for ULC microgel suspensions with ζ ≥ 5.71 ± 0.01.

**Figure 3:**
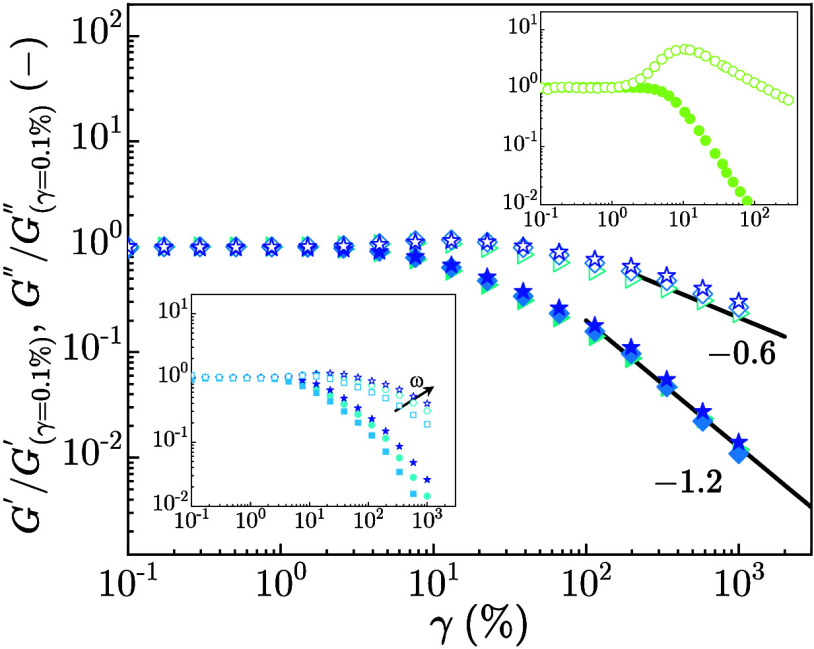
Normalized storage (*G*′, filled) and loss modulus (*G*″, open colored symbols) with the modulus in the linear viscoelastic regime (*G*_γ=0.1%_^′^), (*G*_γ=0.1%_^″^) respectively, versus shear strain amplitude γ (%) for ULC microgel suspensions at ζ = 5.71 ± 0.01 (right triangles), ζ = 7.03 ± 0.01 (diamonds), ζ = 8.16 ± 0.01 (stars). Bottom inset: Normalized (*G*′, filled) and (*G*″, open colored symbols) with (*G*_γ=0.1%_^′^) and (*G*_γ=0.1%_^″^), respectively, versus γ (%) for ULC microgel suspensions at ζ = 8.16 ± 0.01 at ω = 0.1, (squares) ω = 1, (circles), and ω = 10 rad/s, (stars). Top inset: Normalized (*G*′, filled) and (*G*″, open circles) with (*G*_γ=0.1%_^′^) and (*G*_γ=0.1%_^″^), respectively, versus γ (%) for RC suspensions at ζ = 0.70 ± 0.03.

Within the linear viscoelastic regime (γ < 10%), both *G*′ and *G*″ remain independent of strain amplitude. Beyond this regime (γ ≳ 10%), the moduli exhibit shear-thinning behavior characterized by power-law decays, *G*′ ∼ γ^–2π^ and *G*″ ∼ γ^–π^, with an exponent π = 0.6 ± 0.05. This scaling behavior is indicative of a single yielding process, consistent with what is reported in the literature for repulsive soft and hard glassy systems.^31,32^

A notable observation is the absence of overshoot in *G*″ with increasing γ (%). This suggests that the loosely cross-linked polymeric shell of ULC microgels aligns in the shear direction and energy dissipates, a behavior reminiscent of that observed in flexible polymer solutions and melts where chain orientation, stretching, and disentanglement effects occurred.^22,30^ This also means that the poorly cross-linked shell is mainly composed of dangling chains, which can be more easily aligned in the flow. Further rheo-SAXS measurements should provide some insights (e.g., critical shear rates, time scales) on how ULC microgels order under flow, although such experiments remain challenging, especially when proving the velocity-velocity gradient plane.^38^ To further assess the universality of the observed strain-thinning behavior, we investigate the strain amplitude dependence of the viscoelastic response at varying oscillation frequencies ω, focusing on the highest ζ studied (bottom inset, Figure 3). At large γ (%), the nonlinear viscoelastic behavior is characterized by pronounced strain-thinning behavior and the absence of an overshoot in *G*″ across the entire ω range. The top inset of Figure 3 presents the normalized *G*′, *G*″ versus γ (%) for RC microgel suspensions at ζ = 0.70 ± 0.03. RC microgel suspensions exhibit the expected strain-thinning response, accompanied by a marked increase in *G*″ with increasing γ (%), indicative of a yielding mechanism typically associated with glassy systems.^24^

Steady shear rheology was employed to further characterize the flow behavior of ULC and RC microgel suspensions. The resulting flow curves, i.e., the steady-state shear stress σ versus the shear strain rate γ̇, are presented in Figure S7. Downward (decreasing γ̇) and immediately upward (increasing γ̇) flow curves (in agreement with LAOS) reveal that thixotropic effects are not observed in the relevant time scales as the two series of data sets virtually coincide (Figure S8). Figure 4 presents the dimensionless flow curve for ULC (blue-shaded data) and RC (green-shaded data) microgel suspensions across a range of ζ, demonstrating a universal response characteristic of each system. The shear stress is normalized by the critical stress σ_*c*_, determined from LAOS measurements (Figure S9). In these experiments, the yield strain and corresponding critical stress were obtained from the intersection of two linear regimes in the stress–strain amplitude curve, representing a transition from linear to nonlinear viscoelastic behavior. The shear strain rate, γ̇, is scaled by the apparent relaxation time, estimated as η_*s*_/*G*_*P*_, where η_*s*_ is the solvent viscosity and *G*_*P*_ the plateau modulus.^31,39^ For RC microgels, *G*_*P*_ is extracted from the plateau value of storage modulus *G*′ obtained in DFS measurements (Figure S3). For ULC microgel suspensions, where no clear plateau in *G*′ is observed, and *G*′ remains frequency dependent, we select an apparent value *G*′_app_ at ω = 100 rad/s. The universal master-curve exhibits a strong γ̇ dependence, characterized by a rapid decrease in σ. At higher γ̇, σ increases following a square-root power-law scaling with γ̇.^31^ We tested the validity of our claim regarding the universal behavior of ULC microgel suspensions and found that this universality is preserved when considering the apparent storage modulus, *G*′_app_ evaluated at different ω, at 0.01 and 1 rad/s (Figure S10). The dashed line in Figure 4 is plotted according to

1 $$\left(\frac{\sigma }{\sigma_{c}}\right)=A{\left(\frac{\mathrm{\gamma ̇}\eta_{s}}{G_{P}}\right)}^{k}+B{\left(\frac{\mathrm{\gamma ̇}\eta_{s}}{G_{P}}\right)}^{m}$$

**Figure 4:**
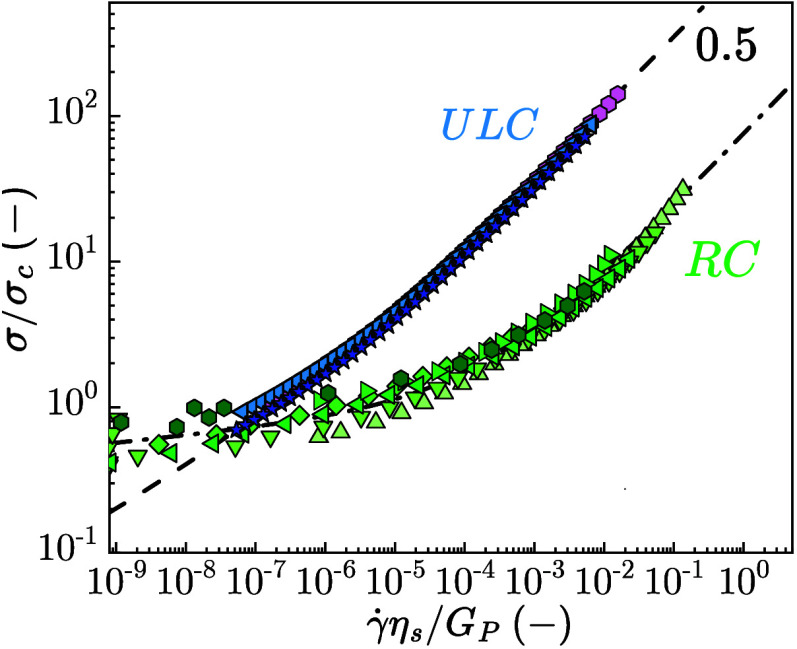
Universal flow curve of normalized shear stress with critical stress σ/σ_*c*_ versus the normalized shear strain rate with the solvent viscosity and plateau modulus, γ̇η_*s*_/*G*_*P*_ of suspensions with ULC microgels at ζ = 5.71 ± 0.01 (polygons), ζ = 7.03 ± 0.01 (left triangles), ζ = 8.16 ± 0.01 (stars) and RC at ζ = 0.6 ± 0.03 (up triangles), ζ = 0.7 ± 0.03 (down triangles), ζ = 0.8 ± 0.03 (diamonds), ζ = 0.9 ± 0.03 (left triangles), ζ = 1.0 ± 0.04 (right triangles), ζ = 1.1 ± 0.04 (pentagons).

At lower γ̇, no clear stress plateau is observed; instead, σ decreases continuously with a power-law exponent *k* = 0.25 ± 0.05, which is notably close to the high γ̇ where shear thinning is observed with an exponent *m* = 0.5 ± 0.05. This flow behavior aligns more closely with the flow behavior of star polymers (σ decreases with γ̇ as a power-law with a weak exponent) than with regular cross-linked, or core–shell microgel suspensions, again reflecting the prominent presence of dangling chains in the poorly cross-linked shell of the ULC microgels.^9,31,32^ It would be of great importance to understand how the length or number of dangling ends is related to the flow behavior. The flow behavior of ULC microgel suspensions also exhibits notable differences compared to viscoelastic polymer solutions. Polymer solutions typically display a pronounced shear thinning behavior for shear rates γ̇ > γ̇_crit_, where γ̇_crit_ is a critical value, and σ ∼ γ̇^–1^ at low γ̇ values.^40^ RC suspensions exhibit the flow behavior characteristic of soft glassy systems. At high γ̇, where σ > σ_*y*_, the master-curve displays a square-root dependence of σ on γ̇, consistent with the Herschel–Buckley model (H–B) with the exponent *n* → 0 (dashed dotted line in Figure 4):

2 $$\sigma =\sigma_{y}+c{\mathrm{\gamma ̇}}^{\beta }$$

where *c* is a prefactor and β = 0.5 ± 0.05 is the power-law exponent. At low γ̇, close to σ ∼ σ_*y*_, RC microgel suspensions exhibit an extended stress plateau, a characteristic feature of microgel suspensions and pastes.^25,39,41^

In summary, we show that these ultrasoft microgels exhibit a fundamentally distinct linear viscoelastic response (*G*′ ∼ *G*″ ∼ ω^*n*^), compatible with critical gel-like dynamics.^23,31^ Their nonlinear viscoelastic behavior, likely driven by arm retraction, shares features with entangled polymer networks, including chain orientation, stretching along the shear direction, and disentanglement effects. We attribute these peculiar rheological properties to the enhanced polymeric contributions arising from a loose polymeric corona with dangling chains and loose cross-linked regions. Furthermore, the strong similarity of the rheological response between our system, flexible polymer networks, and computer simulations of ULC microgels indicates that these microgels are prone to interpenetration.^42^ These observations highlight a clear structure–property relationship, guiding for designing microgels with tunable rheology and motivating comparisons with similar star-like microgels or with single chain nanoparticles (SCN).^43,44^ Indeed, similar to microgels, in SCN, the internal architecture (cross-linker amount) dictates the structural and rheological response to crowding.^45,46^ The main structural difference between SCN and ULC microgels is that ULC microgels have a core, and this provides the possibility to compare how crowding affects the rheological response of systems with similar softness and different internal architecture.^47^ Furthermore, analysis of *G*_*P*_, γ_yield_, σ_*y*_ and their evolution with ζ (Figure S11), suggests that, under specific conditions, we can synthesize microgels characterized by an extremely soft interaction potential. This observation motivates further investigation of the rheological response of comparable soft systems, such as ULC microgels synthesized with different surfactants or monomers, as well as SCN.

Due to their ability to disperse in water and facile large-scale synthesis via straightforward precipitation polymerization, ULC microgels are suitable not only for further investigating stress-relaxation mechanisms in densely packed soft repulsive suspensions without the use of organic or toxic solvents, but also for applications that prioritize sustainability and biocompatibility, such as bioinks and drug delivery systems.^48^

Given their small size, the microgels presented here are also suitable to be used to advance our understanding of more general and biorelevant phenomena. One can think to realize composite hydrogels, using, for instance, matrices of biorelevant polymers (e.g., collagen or gelatin),^49–51^ and incorporating microgels to tune their bulk and interfacial properties. By systematically changing the softness, shape, and interactions of the embedded microgels and the nature of the polymeric scaffold, one can better understand how the interplay between the properties of the matrix and those of the embedded colloids determines the macroscopic properties of a more complex system (e.g., cell).

## Supplementary Material



## Notes

Additional experimental details; Microgel synthesis and characterization; Determination of generalized packing fraction, ζ; SAXS intensity profiles and rheological measurements (DSS, flow curves, DFS) (PDF)
