# Supplementary material for: Ultra-Low-Cross-Linked Microgels Reveal Unexpected Dynamics in Overcrowded Conditions
Source: ACS Macro Lett. 2026 Feb 18;15(3):387–92. doi: 10.1021/acsmacrolett.5c00787 (PMC13001104; doi:10.1021/acsmacrolett.5c00787)
Supplement: Supplementary file 1 [file mz5c00787_si_001.pdf]

Supporting Information: Ultra-Low-Crosslinked microgels reveal unexpected dynamics in overcrowded conditions

Nikolaos A. Burger,[*,†] Alexander V. Petrunin,[‡] Ann E. Terry,[¶] and Andrea Scotti[*,†]

†*Division of Physical Chemistry, Lund University, SE-22100 Lund, Sweden*

‡*Institute of Physical Chemistry, RWTH Aachen University, 52056 Aachen, Germany*

¶*MAX IV Laboratory, Lund University, P.O. Box 118, 22100 Lund Sweden*

E-mail: nikolaos.bourker@chem.lu.se; andrea.scotti@fkem1.lu.se

Contents

I. Synthesis

II. Dynamic Light Scattering

III. Viscosimetry and definition of the generalized packing fraction ζ

IV. Small-angle X-ray Scattering

V. Shear rheology

VI. Evolution of G_P, γ_{yield}, σ_y with ζ.

I. Synthesis

For the synthesis of ultralow-crosslinked (ULC) microgels, NIPAM (2.3769 g, 0.07 M) and sodium dodecyl sulfate (0.1730 g, 2 mM), SDS, were dissolved in 295 mL double-distilled H_2O that was filtered through a 0.2 μm syringe filter. The solution was brought to 70°C in a three-neck flask. Potassium persulfate (0.1265 g, 1.56 mM), KPS, was dissolved in 5 mL of the same double-distilled H_2O. Both solutions were bubbled with N_2 for 45 min, after which the reaction was started by adding the KPS into the flask. The reaction mixture was kept at 70°C under N_2 flow for 4.5 h. The microgels were purified by five-fold ultra-centrifugation at 50,000 rpm followed by redispersion in fresh double-distilled water. Freeze-drying was used for storage. We performed this synthesis twice and in both cases microgels with a hydrodynamic radius of 114 ± 2 have been obtained with comparable swelling ratio, i.e. softness. This shows the reproducibility of this precipitation polymerisation approach to obtain small ultra-soft microgels. For the synthesis of RC microgels, NIPAM (5.1607 g, 0.152 M), N,N'-methylenebisacrylamide) (0.2714 g, 0.006 M), BIS, and SDS (0.1081 g, 1.25 mM) were dissolved in 295 mL double-distilled H_2O that was filtered through a 0.2 µm syringe filter. The solution was brought to 70°C in a large flask. KPS (0.1265 g, 1.56 mM) was dissolved in 5 mL of the same double-distilled H_2O. Both solutions were bubbled with N2 for 60 min, after which the reaction was started by adding the KPS into the flask. The reaction mixture was kept at 70°C under N_2 flow for 4 h. The microgels were purified by five-fold ultra-centrifugation at 40,000 rpm followed by redispersion in fresh double-distilled water. Freeze-drying was used for storage.

II. Dynamic Light Scattering

The dynamic light scattering (DLS) measurements were performed on a Mod3D-DLS

Spectrometer (LS Instruments, Switzerland) equipped with a 660 nm Cobolt laser with a maximum power of 100 mW. 5 mm cylindrical glass cells were used and placed in the temperature-controlled index-matching bath containing decalin. The fluctuations of the scattered light were detected within a scattering angle range between 20° and 135° with 5° steps by avalanche photodiodes and processed by an LS Instrument correlator.

The average decay rate, Γ, was obtained with cumulant analysis. The decay rates depend on the scattering vector *via* the average diffusion coefficient $D_0 : \Gamma = D_0 q^2$. The average diffusion coefficient was obtained by plotting Γ versus q^2 and fitting the data with linear regression. Finally, the Stokes-Einstein relation was used to obtain the hydrodynamic radius $R_h : R_h = k_B T/(6\pi\eta D_0)$ with k_B and η the Boltzmann constant and the viscosity of water, respectively.

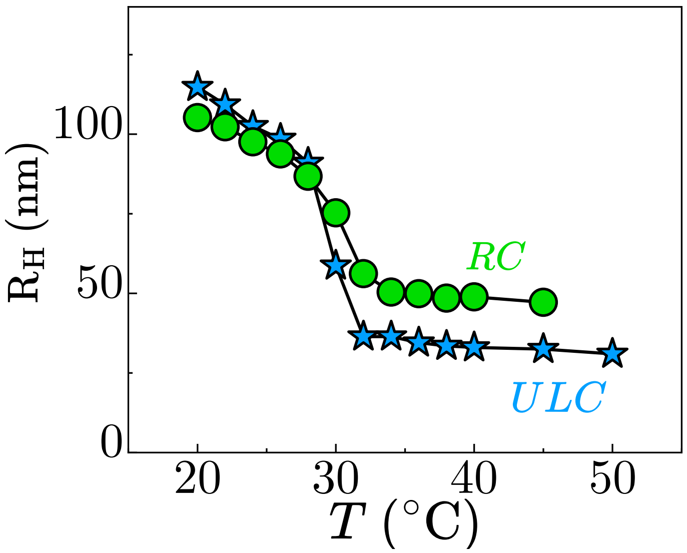

Figure S1: Hydrodynamic radius derived from dynamic light scattering measurements of dilute ($\zeta = 0.08$) ULC (stars) and RC (circles) microgel suspensions versus temperature T.

III. Viscosimetry and definition of the generalized packing fraction ζ

To maintain a common language with respect to hard spheres, whose phase behavior is

determined by their volume fraction in suspension, ϕ, one can define a generalized packing fraction, $\zeta = \frac{Nv_0}{V}$, when using soft colloids.[S1,S2] Here, N is the number of particles in suspension, v_0 is their volume in the swollen state measured in dilute conditions well below their volume phase transition temperature, and V is the total volume of the suspension. In the limit of highly diluted samples, at low packing fractions, the course of η_r, the solution viscosity divided by the solvent viscosity, as a function of the sphere packing fraction ϕ is well described by the Einstein-Batchelor equation:

$$\eta_r = 1 + 2.5\phi + 5.9\phi^2 \tag{1}$$

This equation also describes the course of viscosity with increasing the concentration of microgels in solution, in the limit of highly diluted samples.[S3,S4] The formal substitution of the real particle volume fraction ϕ with the generalized volume fraction ζ, $\phi = \zeta$, is valid at low concentrations where the microgels do not experience any deswelling or deformation, allowing us to rewrite Eq. 2:

$$\eta_r = 1 + 2.5\zeta + 5.9\zeta^2 \,. \tag{2}$$

The value of ζ is related to the microgel weight fraction in solution, c, by means of a multiplicative constant, k.[S4–S12] Substituting $\zeta = kc$ in the previous equation leads to $\eta_r = 1 + 2.5kc + 5.9(kc)^2$. This relation is used to fit the data of the relative viscosity of the solution of RC and ULC microgel suspensions. From these fits, the value of k is determined and then used to obtain the values of ζ used in our study.

IV. Small-Angle X-ray Scattering

The SAXS intensities for static samples, contained in quartz capillaries (Hilgenberg) with 1.5 mm diameter and wall thickness of 0.1 mm have been measured at the CoSAXS

beamline at the 3 GeV ring of the MAX-IV Laboratory (Lund, Sweden). The q-range of interest between $7{\cdot}10^{-3}$ and 0.7 nm^{-1} was covered on CoSAXS using a sample-to-detector distance of 14.2 m with x-ray beam energy E = 12.4 keV. The instrument is equipped with an Eiger2 4M SAXS detector with a pixel size of 75 μm x 75 μm. A Python-based code was used to convert the 2D images to 1D profiles.

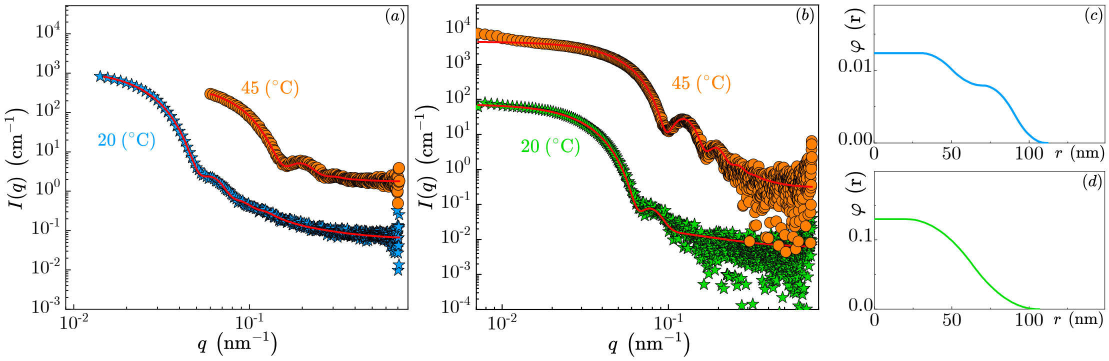

Figure S2: Small-angle X-ray scattering intensities $I(q)$ versus scattering vector q of (a) ULC and (b) RC microgel suspensions measured at 20 °C (stars) and 45 °C (circles). The top curves at 45 °C are shifted vertically by a factor of 10^2 for clarity. Solid lines: fits of the data with a fuzzy-sphere model.[S13] Relative polymer radial distribution as obtained from the fit of the form factor at 20 °C for (c) ULC and (d) RC microgel suspensions.

Fig. S2(a) shows the SAXS intensity profiles $I(q) \simeq P(q)$ of dilute ULC microgels suspension ($\zeta = 0.08$) measured in swollen $T = 20$ °C (stars) and collapsed state 45 °C (circles), respectively. The black solid lines represent the fits of the data with a core-fuzzy-shell model[S14] which accounts for the possibility of having a core with a different scattering length density with respect to the less dense shell. The total radius of the ULC microgels is (111 ± 4) nm at 20 °C and (27 ± 2) nm at 45 °C. This model was necessary since fitting the data with the common fuzzy-sphere models,[S13] leads to an unreliably high total radius compared to the R_H obtained from dynamic light scattering (DLS).

Fig. S2(b) shows the data relative to SAXS measurements of dilute RC microgels suspension ($\zeta = 0.08$) at 20 °C (stars) and 45 °C (circles). The solid line is a fit with the simple fuzzy-sphere model since, in contrast to the case of the ULC microgel, the use of this

simpler model leads to both a good fit and a value of the total radius comparable to the hydrodynamic radius measured with DLS. We note that, when we use the model in Eqs. 3 and 4, we obtain the same radial distribution using the simple fuzzy-sphere model.

The fuzzy core-shell model consists of an interpenetrating layer of core and shell. The length of this layer is $2\sigma_{in}$. The outer surface is also characterized by a decreasing polymer density. The length of this region is σ_{out}. The widths of the inner and outer regions with constant density are W_{core} and W_{sh}, respectively. For a particle with such a radial distribution, one can write the scattering amplitude $A(q)$ as:

$$\begin{aligned} A(q) &= \Delta\rho_{sh} V_{sh} \Phi_{sh}(q, R_{out}, \sigma_{out}) \\ &+ (\Delta\rho_{core} - \Delta\rho_{sh}) V_{core} \Phi_{core}(q, R_{in}, \sigma_{in}) \end{aligned} \tag{3}$$

where $\Delta\rho$ is the difference between the scattering length density of the solvent and the core (or the shell). V_{core} and V_{sh} are the core and shell volumes, respectively. The radii are defined as $R_{in} = W_{core} + \sigma_{in}$, $R_{out} = W_{core} + 2\sigma_{in} + W_{sh} + \sigma_{out}$ and the total radius is $R = R_{in} + R_{out}$. The normalized Fourier transform of the radial density profile can be written as follows:

$$\begin{aligned} \Phi(q, R, \sigma) &= \frac{1}{V_n}\Big[\left(\frac{R}{\sigma^2} + \frac{1}{\sigma}\right)\frac{\cos[q(R+\sigma)]}{q^4} \\ &+ \left(\frac{R}{\sigma^2} - \frac{1}{\sigma}\right)\frac{\cos[q(R-\sigma)]}{q^4} - \frac{3\sin[q(R-\sigma)]}{q^5\sigma^2} \\ &- \frac{2R\cos(qR)}{q^4\sigma^2} + \frac{6\sin(qR)}{q^5\sigma^2}\Big] \end{aligned} \tag{4}$$

where $V_n = R^3/3 + R\sigma^2/6$.[S15] The model is then convoluted with a Gaussian to account for the particle size polydispersity p and for the SANS data with the resolution function of the instrument. Finally, a Lorentzian term is added to account for the high-q part of the

data:

$$I_L(q) = \frac{I_L(0)}{1 + q^2\xi^2}\,, \tag{5}$$

where $I_L(0)$ is the contribution of the Lorentzian term at $q = 0$, and ξ is the average mesh size of the polymeric network, or in other words, the average distance between two crosslinked points. The model we use in Eqs. 3-4 is obtained assuming a radial distribution for a fuzzy-core shell particles with different densities for the core and the shell. The fits of the data with the model give us the values of all the parameters needed to draw the radial profile. Therefore, the radial profile of the polymer within the particle $\varphi(r)$ is calculated using the characteristic lengths obtained from fitting the data with Eqs. 3 and 4.

V. Shear rheology

We used an MCR Anton Paar 501 stress-controlled rheometer. Measurements performed with stainless steel cone-plate geometries with smooth surfaces (cone angle 1°) and two different cone diameters at 25 and 50 mm, respectively. All measurements were performed at 20 °C. A home-made solvent trap was employed to minimize evaporation. At the end of the experiment, we performed short tests to ensure no evaporation. Although we didn't observe signatures of aging or shear-history effects, we follow a strict shear protocol for consistency. The rejuvenation protocol applied before any oscillatory shear measurement consisted of (i) the application of $\dot{\gamma} = 1000\ s^{-1}$ for 60 seconds and (ii) shear cessation, where we follow the evolution of G', G'' at $\omega = 1\ rad/s$ for a certain period of time (300 seconds). For steady shear experiments, measurements were performed immediately after the application of $\dot{\gamma} = 1000\ s^{-1}$ for 60 seconds, with a steady shear rate sweep (flow curve) initially with decreasing $\dot{\gamma}$ and subsequently upon increasing $\dot{\gamma}$.[S16,S17] Repeatable LAOS and flow curves experiments do not show any signature of hysteresis, as the subsequent datasets

coincide very well.

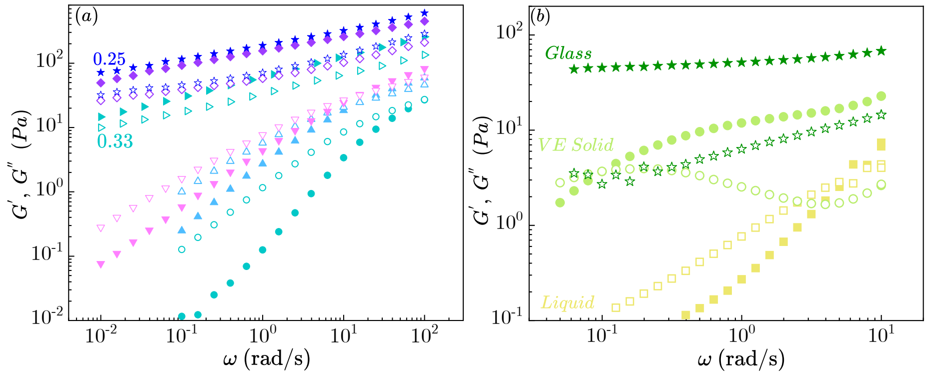

Figure S3: Evolution of loss G'' (open) and storage modulus G' (filled) versus frequency ω after rejuvenation for suspensions of (a) ULC at $\zeta = 3.06 \pm 0.01$ (circles), $\zeta = 3.67 \pm 0.01$ (up triangles), $\zeta = 4.48 \pm 0.01$ (down triangles), $\zeta = 5.71 \pm 0.01$ (right triangles), $\zeta = 7.03 \pm 0.01$ (diamonds), $\zeta = 8.16 \pm 0.01$ (stars) and (b) RC at $\zeta = 0.6 \pm 0.03$ (squares), $\zeta = 0.65 \pm 0.03$ (circles), $\zeta = 0.8 \pm 0.03$ (stars).

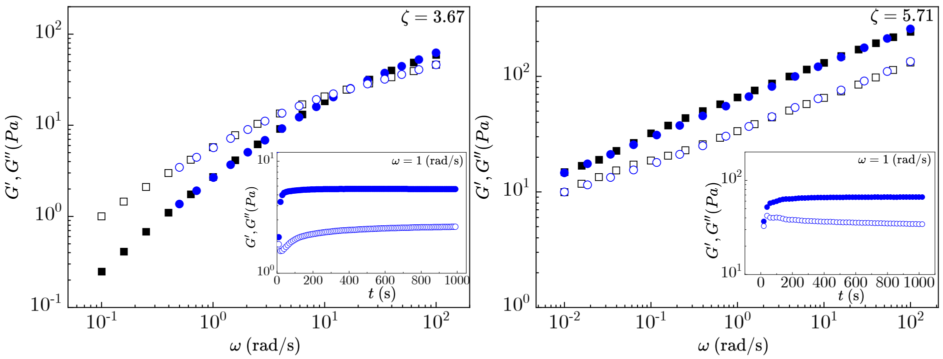

Figure S4: Evolution of loss G'' (open) and storage modulus G' (filled) versus frequency, ω for suspensions of (a) ULC at (a) $\zeta = 3.67 \pm 0.01$ and (b) $\zeta = 5.71 \pm 0.01$. Squares and circles indicate repeatable measurements to test for possible slow evolution after rejuvenation. *Inset:* Evolution of G', G'' with time upon application of $\gamma = 1\%$ after rejuvenation and prior to DFS measurements.

From the dimensionless analysis of oscillatory frequency sweep measurements, we find that the crossover point between the G' and G'', occurs at very high generalized Peclet number, P_{e_o}, where $P_{e_o} = \omega R_h^2/D = \omega R_h^3(6\pi\eta_0/k_BT)$ and compares the frequency, ω, with the time, τ, an isolated microgel requires to diffuse a distance identical to its radius in a medium with zero-shear viscosity η_0.[S18] At $P_{e_o} = \omega\tau = 1$, concentrated suspensions exhibit

a predominantly viscous, liquid-like behavior, as evidenced by $G'' > G'$ indicating rapid stress relaxation dynamics. A solid-like response is observed at $P_{e_o} \sim 1000$, one order of magnitude higher than those reported for larger ULC and regular crosslinked microgels where $P_{e_o} = 100$.[S19] This observation further suggests that, even below the dynamic arrest, the viscoelastic response is significantly influenced by the contributions of the extremely soft polymeric shell and dangling chains of the individual microgels.

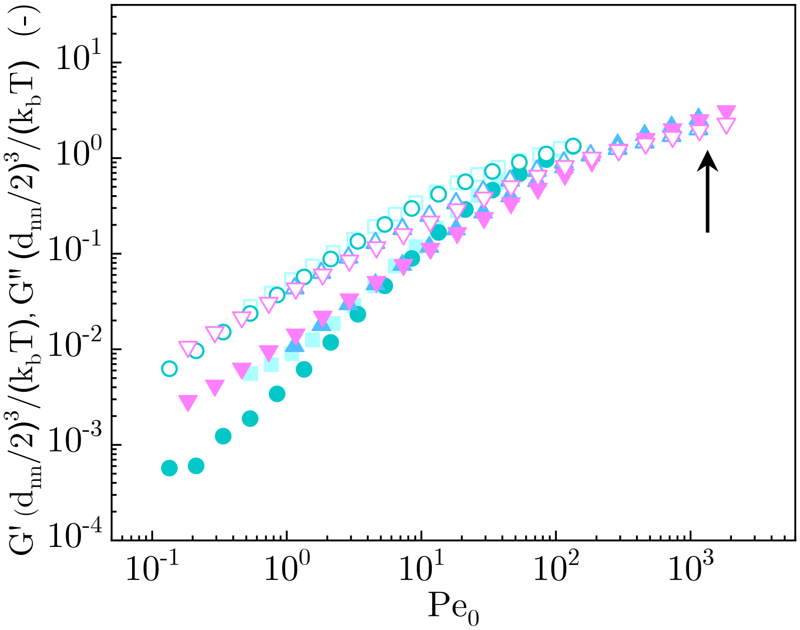

Figure S5: Rescaled loss G'' (open) and storage modulus G' (filled) with thermal energy per particle versus P_{e_o} for suspensions of ULC at $\zeta = 2.5 \pm 0.01$ (squares), $\zeta = 3.06 \pm 0.01$ (circles), $\zeta = 3.67 \pm 0.01$ (up triangles), $\zeta = 4.48 \pm 0.01$ (down triangles).

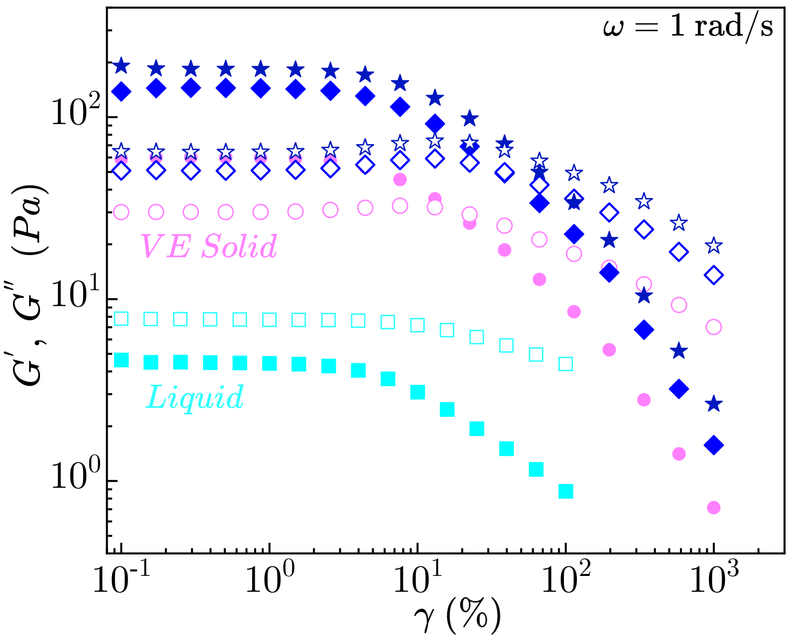

Figure S6: Evolution of loss G'' (open) and storage modulus G' (filled) with shear strain amplitude γ (%) performed from low to high γ (%) after rejuvenation, for ULC microgel suspensions at $\zeta = 4.48 \pm 0.01$ (squares), $\zeta = 5.71 \pm 0.01$ (circles), $\zeta = 7.03 \pm 0.01$ (diamonds), $\zeta = 8.16 \pm 0.01$ (stars).

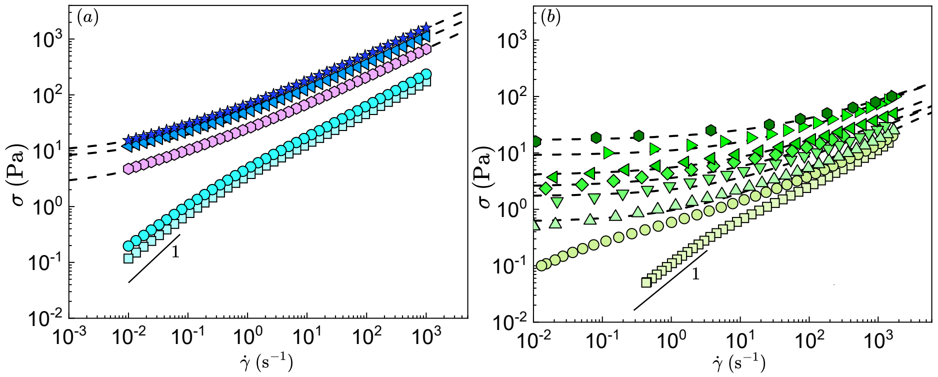

Figure S7: Evolution of stress σ versus shear strain rate, $\dot{\gamma}$ performed from high to low $\dot{\gamma}$ after rejuvenation for suspensions of (a) ULC at $\zeta = 3.67 \pm 0.01$ (squares), $\zeta = 4.48 \pm 0.01$ (circles), $\zeta = 5.71 \pm 0.01$ (polygons), $\zeta = 7.03 \pm 0.01$ (left triangles), $\zeta = 8.16 \pm 0.01$ (stars) and (b) RC at $\zeta = 0.6 \pm 0.03$ (squares), $\zeta = 0.65 \pm 0.03$ (circles), $\zeta = 0.7 \pm 0.03$ (up triangles), $\zeta = 0.75 \pm 0.03$ (down triangles), $\zeta = 0.8 \pm 0.03$ (diamonds), $\zeta = 0.9 \pm 0.03$ (left triangles), $\zeta = 1 \pm 0.04$ (right triangles), $\zeta = 1.1 \pm 0.04$ (polygons). Dashed lines are fit of the data with the H-B model: $\sigma = \sigma_y + c\,\dot{\gamma}^{\beta}$. Solid lines depict the low shear slope ($\sigma \sim \dot{\gamma}$) of the data observed at lower ζ.

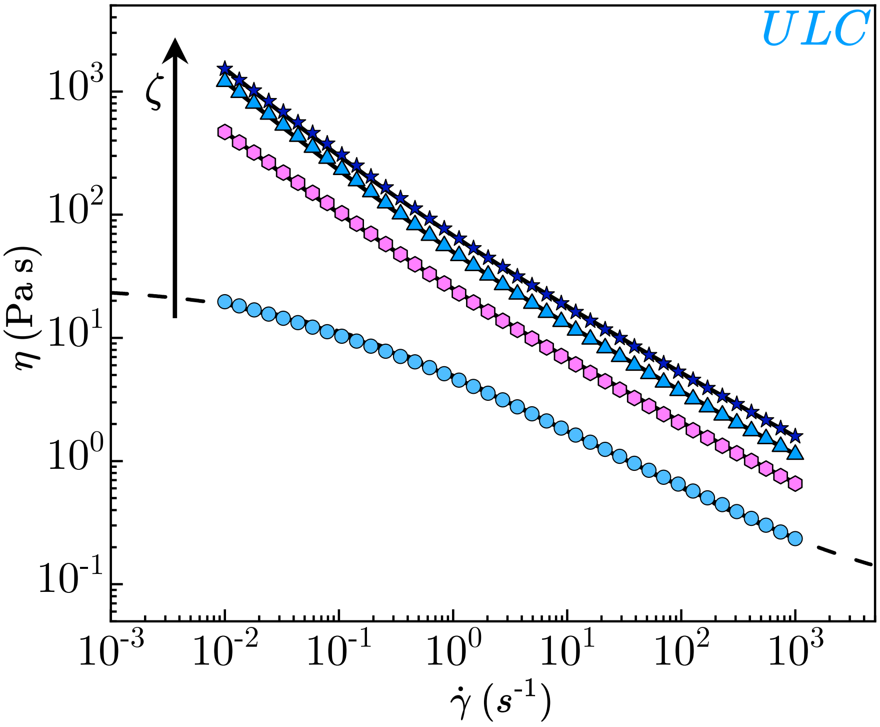

Figure S8: Evolution of viscosity η versus shear strain rate, $\dot{\gamma}$ for suspensions of ULC performed from high to low (symbols) and low to high (lines) $\dot{\gamma}$ after rejuvenation at $\zeta = 4.48 \pm 0.01$ (circles), $\zeta = 5.71 \pm 0.01$ (polygons), $\zeta = 7.03 \pm 0.01$ (up triangles), $\zeta = 8.16 \pm 0.01$ (stars). Dashed line represent fit of the data with: $\frac{\eta-\eta_0}{\eta_0-\eta_\infty} = \frac{1}{\left(1+\frac{\gamma}{\gamma_c}\right)^m}$, where m the power-law exponent counts for shear-thinning behavior, η and η_∞ the apparent and infinite shear viscosity, and γ and γ_c the strain and critical strain rate, respectively.

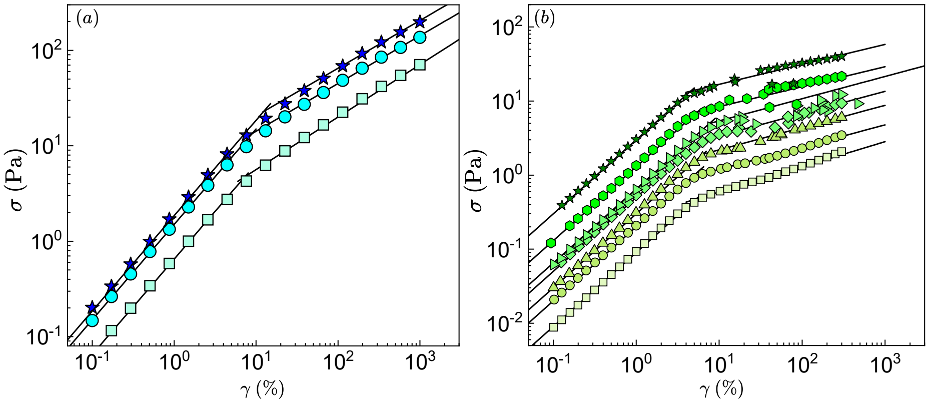

Figure S9: Stress, σ versus strain amplitude γ (%) for suspensions of (a) ULC at $\zeta = 5.71 \pm 0.01$ (squares), $\zeta = 7.03 \pm 0.01$ (circles), $\zeta = 8.16 \pm 0.01$ (stars) and (b) RC at $\zeta = 0.6 \pm 0.03$ (squares), $\zeta = 0.65 \pm 0.03$ (circles), $\zeta = 0.7 \pm 0.03$ (up triangles), $\zeta = 0.8 \pm 0.03$ (diamonds), $\zeta = 0.9 \pm 0.03$ (right triangles), $\zeta = 1 \pm 0.04$ (polygons), $\zeta = 1.1 \pm 0.04$ (stars). For clarity, only measurements from low to high γ (%) are shown. We didn't observe any signature of hysteresis as the curves coincide very well.

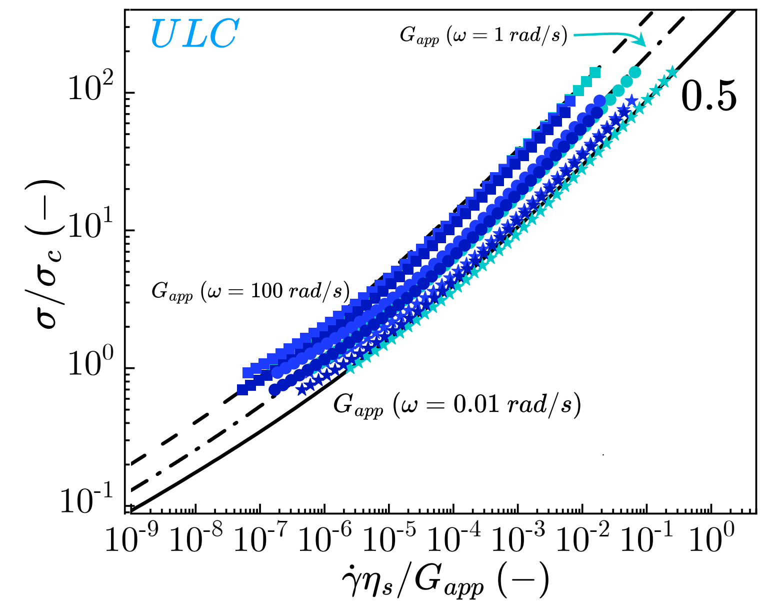

Figure S10: Universal flow curve of normalized shear stress with critical stress σ/σ_c versus the normalized shear strain rate with the solvent viscosity and apparent plateau modulus, $\dot{\gamma}\eta_s/G_{app}$ of ULC microgel suspensions at $\zeta = 5.71 \pm 0.01$ (cyan), $\zeta = 7.03 \pm 0.01$ (blue), $\zeta = 8.16 \pm 0.01$ (navy) for G_{app} defined at $\omega = 0.01$ (stars), $\omega = 1$ (circles) and $\omega = 100$ (squares) rad/s.

VI. Evolution of G_P, γ_{yield}, σ_{yield} with ζ.

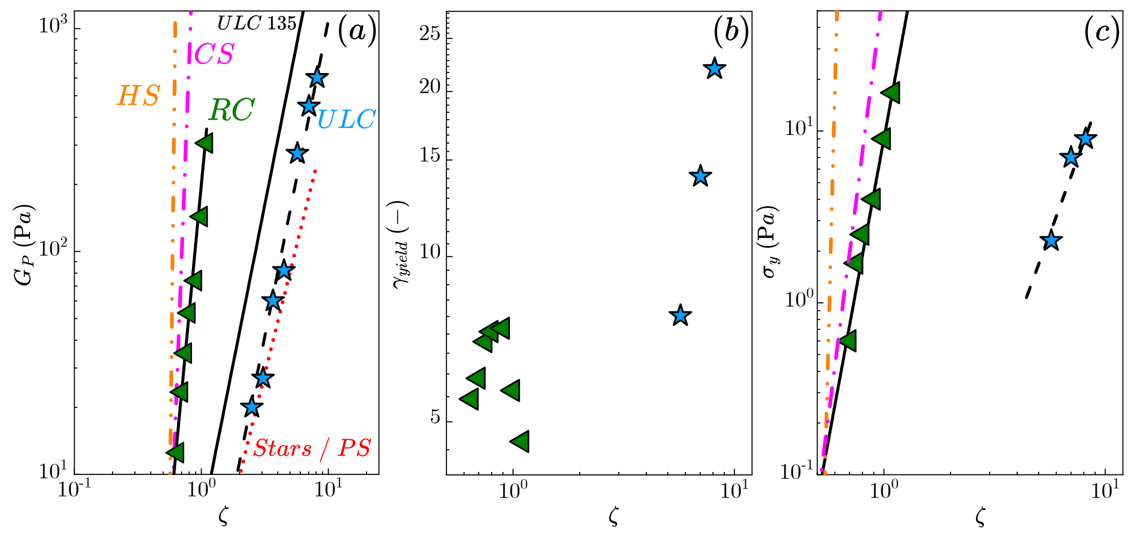

Figure S11: Evolution of (a) plateau modulus, (G_P), (b) yield strain, (γ_{yield}) and (c) yield stress, (σ_y) with (ζ) of ULC (stars) and RC (triangles) microgel suspensions. Dashed double dot line is the power law evolution of hard sphere glasses,[S20] dashed dot line is attributed to core-shell particles,[S20] full line to larger and stiffer ULC microgels[S19] and dots are attributed to low-arm stars and polymer solutions.[S21]

Figure S11 presents the evolution of the plateau modulus, G_P, yield strain, γ_{yield}, and yield stress σ_y versus the generalized packing fraction ζ for ULC (stars) and RC (triangles) microgel suspensions.

In Figure S11(a), G_P is plotted against ζ. For ULC microgel suspensions where no clear G_P is observed, we use for G_P the apparent values derived from DFS (G' value at $\omega = 100$ rad/s, where $G' > G''$). ULC microgel suspensions exhibit a significantly weaker concentration dependence of elasticity compared to RC microgels with comparable size; such a behavior is captured by Eq. 6:

$$G_P = a \cdot \zeta^{1+\frac{\lambda}{3}} \tag{6}$$

Notably, for ULC microgel suspensions $\lambda = 5.5 \pm 0.2$; this value is much lower compared to the one observed in suspensions of RC microgels, where $\lambda = 15.0 \pm 0.2$. This low λ

value also contrasts sharply with those reported for other cross-linked microgels,[S22] larger ULC microgels,[S19] and multi-arm stars.[S23] In contrast, such a low value of λ aligns well with the results obtained for flexible polymer networks.[S21,S24,S25] Since low values of λ indicate a softer particle-particle interaction potential,[S2,S4,S19] this once more highlights that the microgel-to-microgel interaction is mainly mediated by the dangling chains surrounding the ULC microgels, which makes the interaction potential much softer compared to other cases reported in the literature.

In Figure S11(b), the γ_{yield} for both ULC and RC microgel suspensions is plotted versus ζ. The values of γ_{yield} are extracted from dynamic strain sweeps (DSS) measurements, based on the intersection point between the low and high strain regimes (Figure S9. ULC microgel suspensions, despite their significantly lower elasticity and the critical-gel-like dynamics at this ζ, demonstrate an ability for large deformation before entering the non-linear regime, attaining a maximum $\gamma_{yield} = 22\%$, which is more than three times higher than that observed for RC microgel suspensions. For RC microgels, γ_{yield} exhibits a non-monotonic dependence on ζ, reaching a maximum value of approximately 7%. Similar behavior has been observed for hard sphere glasses and cross-linked microgels.[S20] Figure S11(c) presents σ_y for ULC and RC microgel suspensions plotted versus ζ. The values are obtained from fitting the steady shear data to the H-B model and shown in Figure S7. ULC microgel suspensions display a weak dependence of σ_y on concentration, $\sigma_y \sim \zeta^{3.5\pm0.2}$. RC microgel suspensions exhibit a pronounced increase in σ_y as the system approaches the glass transition at ($\zeta = 0.70\pm0.03$), where they behave as weak yield-stress fluids with $\sigma_y \sim 1\ Pa$. Within the glassy regime, σ_y increases sharply and follows a power-law scaling with concentration, $\sigma_y \sim \zeta^{8.0\pm0.2}$, compatible with the behavior of soft colloidal glasses.[S12,S22]

References

(S1) Scotti, A. Characterization of the volume fraction of soft deformable microgels by means of small-angle neutron scattering with contrast variation. *Soft Matter* **2021**, *17*, 5548–5559.

(S2) Scotti, A.; Schulte, M. F.; Lopez, C. G.; Crassous, J. J.; Bochenek, S.; Richtering, W. How Softness Matters in Soft Nanogels and Nanogel Assemblies. *Chemical Reviews* **2022**, *122*, 11675–11700.

(S3) Borrega, R.; Cloitre, M.; Betremieux, I.; Ernst, B.; Leibler, L. Concentration dependence of the low-shear viscosityof polyelectrolyte micro-networks: From hard spheresto soft microgels. *Europhysics Letters* **1999**, *47*, 729.

(S4) Senff, H.; Richtering, W. Temperature sensitive microgel suspensions: Colloidal phase behavior and rheology of soft spheres. *J. Chem. Phys.* **1999**, *111*, 1705–1711.

(S5) Gasser, U.; Liétor-Santos, J.-J.; Scotti, A.; Bunk, O.; Menzel, A.; Fernandez-Nieves, A. Transient formation of bcc crystals in suspensions of poly (N-isopropylacrylamide)-based microgels. *Physical Review E* **2013**, *88*, 052308.

(S6) Gasser, U.; Hyatt, J.; Lietor-Santos, J.-J.; Herman, E.; Lyon, L. A.; Fernandez-Nieves, A. Form factor of pNIPAM microgels in overpacked states. *The Journal of chemical physics* **2014**, *141*.

(S7) Scotti, A.; Gasser, U.; Herman, E. S.; Pelaez-Fernandez, M.; Han, J.; Menzel, A.; Lyon, L. A.; Fernández-Nieves, A. The role of ions in the self-healing behavior of soft particle suspensions. *Proceedings of the National Academy of Sciences* **2016**, *113*, 5576–5581.

(S8) Scotti, A.; Gasser, U.; Herman, E.; Han, J.; Menzel, A.; Lyon, L. A.; Fernandez-Nieves, A. Phase behavior of binary and polydisperse suspensions of compressible

microgels controlled by selective particle deswelling. *Physical Review E* **2017**, *96*, 032609.

(S9) Mohanty, P. S.; Nöjd, S.; Gruijthuijsen, K. v.; Crassous, J. J.; Obiols-Rabasa, M.; Schweins, R.; Stradner, A.; Schurtenberger, P. Interpenetration of polymeric microgels at ultrahigh densities. *Scientific Reports* **2017**, *7*, 1487.

(S10) Conley, G. M.; Aebischer, P.; Nöjd, S.; Schurtenberger, P.; Scheffold, F. Jamming and overpacking fuzzy microgels: Deformation, interpenetration, and compression. *Science Advances* **2017**, *3*, e1700969.

(S11) Conley, G. M.; Zhang, C.; Aebischer, P.; Harden, J. L.; Scheffold, F. Relationship between rheology and structure of interpenetrating, deforming and compressing microgels. *Nature Communications* **2019**, *10*, 2436.

(S12) Pellet, C.; Cloitre, M. The glass and jamming transitions of soft polyelectrolyte microgel suspensions. *Soft matter* **2016**, *12*, 3710–3720.

(S13) Stieger, M.; Richtering, W.; Pedersen, J. S.; Lindner, P. Small-angle neutron scattering study of structural changes in temperature sensitive microgel colloids. *The Journal of chemical physics* **2004**, *120*, 6197–6206.

(S14) Berndt, I.; Pedersen, J. S.; Richtering, W. Temperature-Sensitive Core–Shell Microgel Particles with Dense Shell. *Angewandte Chemie* **2006**, *118*, 1769–1773.

(S15) Scotti, A.; Bochenek, S.; Brugnoni, M.; Fernandez-Rodriguez, M.-A.; Schulte, M. F.; Houston, J.; Gelissen, A. P.; Potemkin, I. I.; Isa, L.; Richtering, W. Exploring the colloid-to-polymer transition for ultra-low crosslinked microgels from three to two dimensions. *Nature Communications* **2019**, *10*, 1–8.

(S16) Burger, N. A.; Loppinet, B.; Clarke, A.; Petekidis, G. Tuning the mechanical proper-

ties of organophilic clay dispersions: Particle composition and preshear history effects. *Journal of Rheology* **2024**, *68*, 695–707.

(S17) Burger, N. A.; Loppinet, B.; Clarke, A.; Petekidis, G. How Preparation Protocols Control the Rheology of Organoclay Gels. *Industrial & Engineering Chemistry Research* **2025**, *64*, 6980–6991.

(S18) Crassous, J. J.; Siebenbürger, M.; Ballauff, M.; Drechsler, M.; Hajnal, D.; Henrich, O.; Fuchs, M. Shear stresses of colloidal dispersions at the glass transition in equilibrium and in flow. *The Journal of chemical physics* **2008**, *128*.

(S19) Scotti, A.; Brugnoni, M.; Lopez, C. G.; Bochenek, S.; Crassous, J. J.; Richtering, W. Flow properties reveal the particle-to-polymer transition of ultra-low crosslinked microgels. *Soft Matter* **2020**, *16*, 668–678.

(S20) Koumakis, N.; Pamvouxoglou, A.; Poulos, A.; Petekidis, G. Direct comparison of the rheology of model hard and soft particle glasses. *Soft Matter* **2012**, *8*, 4271–4284.

(S21) Rubinstein, M.; Colby, R. H. *Polymer physics*; Oxford university press, 2003.

(S22) Ghosh, A.; Chaudhary, G.; Kang, J. G.; Braun, P. V.; Ewoldt, R. H.; Schweizer, K. S. Linear and nonlinear rheology and structural relaxation in dense glassy and jammed soft repulsive pNIPAM microgel suspensions. *Soft matter* **2019**, *15*, 1038–1052.

(S23) Erwin, B. M.; Cloitre, M.; Gauthier, M.; Vlassopoulos, D. Dynamics and rheology of colloidal star polymers. *Soft Matter* **2010**, *6*, 2825–2833.

(S24) Burger, N. A.; Meier, G.; Bouteiller, L.; Loppinet, B.; Vlassopoulos, D. Dynamics and Rheology of Supramolecular Assemblies at Elevated Pressures. *The Journal of Physical Chemistry B* **2022**, *126*, 6713–6724.

(S25) Shahid, T.; Huang, Q.; Oosterlinck, F.; Clasen, C.; Van Ruymbeke, E. Dynamic

dilution exponent in monodisperse entangled polymer solutions. *Soft Matter* **2017**, *13*, 269–282.
